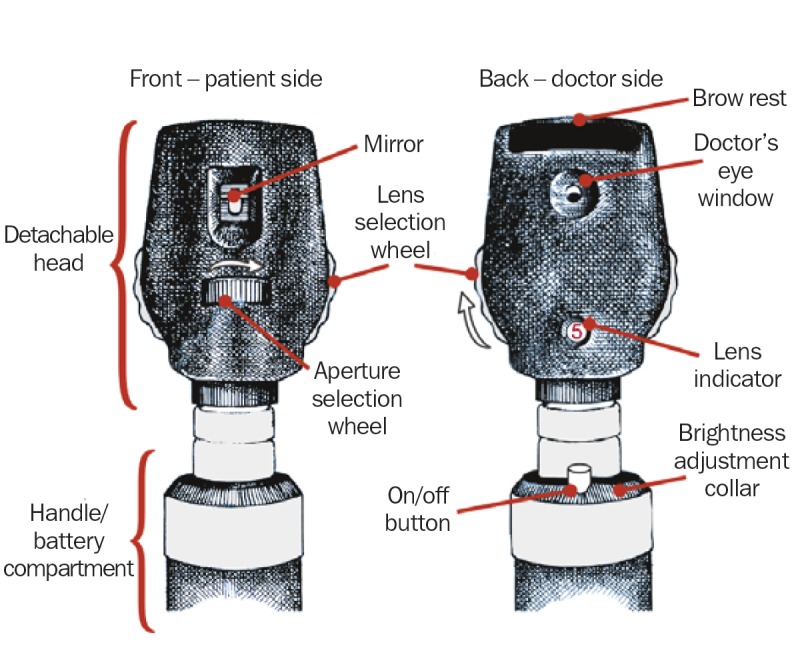# Understanding and caring for the direct ophthalmoscope

**Published:** 2016

**Authors:** Ismael Cordero

**Affiliations:** Clinical Engineer, Philadelphia, USA. **ismaelcordero@me.com**

**Figure:**
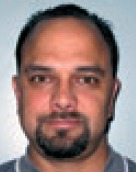
Ismael Cordero

A direct ophthalmoscope, or simply an ophthalmoscope, is a hand-held optical instrument used to inspect the fundus or back of the eye.

The ophthalmoscope (Figure 1) contains a handle with a rechargeable battery and a head, frequently detachable, that contains a bulb, a set of apertures for the light source, and a set of lenses. The view provided by the ophthalmoscope is monocular, non-stereoscopic (2D), narrow field (5°), and is magnified about 15 times.

Light from a bulb (Figure 2) is reflected at right angles and projected as a spot through the iris of the patient to illuminate the retina. This reflection is achieved using a mirror or prism. The illuminated retina is seen directly by the health professional (the user) through the iris of the patient.

The ophthalmoscope can be adjusted to suit the task at hand. A disc or wheel contains lenses of different powers and the required lens can be brought into the line of sight to correct any refractive error on the part of the patient (or of the user if she is not using her spectacles). The user looks just above the mirror or reflecting prism. Many ophthalmoscopes include a set of filters to cut out reflection from the cornea orto reduce the red glare from the retina. A disk or wheel allows the user to change the aperture of the light source. A small aperture is used for an undilated or small pupil. A regular aperture is used otherwise. A slit aperture is used as in a slit lamp. Finally, the brightness of the light can be adjusted by rotating the collar surrounding the on/off button.

## Care

- Keep the instrument in its case or pouch when not in use.
- Make sure the on-off switch is fully turned off (a click sound will be heard) before placing the instrument in its case.
- Recharge the batteries by placing the ophthalmoscope handle in the charging base at the end of each working day.
- When the ophthalmoscope is not likely to be used for long periods of time, remove the batteries from the handle to avoid leakage.
- Wipe dust off the outside of the instrument daily.
- While storing the instrument, keep the lens disc on the zero setting so dust does not build up on the other lenses (the zero setting is just a hole with no lens).
- Some ophthalmoscopes include a shutter for the viewing window. It should be closed when the instrument is not in use to prevent dust from entering.

**Figure 1:**
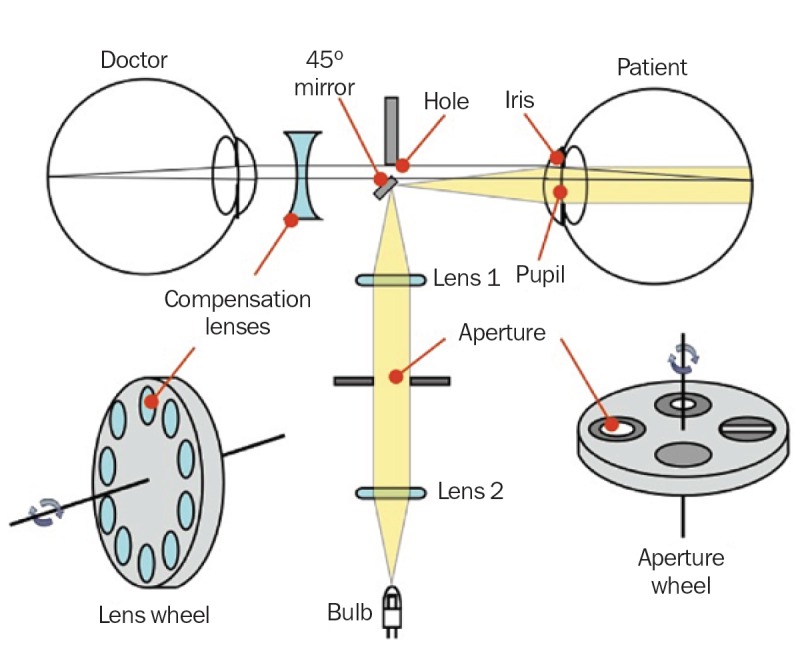


**Figure 2:**